# Substrate specificity-enabled terminal protection for direct quantification of circulating MicroRNA in patient serums†
†Electronic supplementary information (ESI) available: Diameter distribution of 30T-P-templated CuNPs, effect of exo I on the fluorescence of 30T-templated CuNPs and 30T-P-templated CuNPs, optimization of exo I in P-induced terminal protection, results of CD measurements for the ALP assay, results of PAGE analysis for the hsa-miR-21-5p biosensor, comparison of different hsa-miR-21-5p biosensors and related references for supporting information. See DOI: 10.1039/c8sc05240a


**DOI:** 10.1039/c8sc05240a

**Published:** 2019-05-01

**Authors:** Junyao Li, Wenxin Fu, Zhaoyin Wang, Zhihui Dai

**Affiliations:** a Jiangsu Collaborative Innovation Center of Biomedical Functional Materials , Jiangsu Key Laboratory of Biofunctional Materials , School of Chemistry and Materials Science , Nanjing Normal University , Nanjing , 210023 , P. R. China . Email: daizhihuii@njnu.edu.cn ; Fax: +86-25-85891051 ; Tel: +86-25-85891051; b Nanjing Normal University Center for Analysis and Testing , Nanjing , 210023 , P. R. China

## Abstract

Currently, reported affinity pairings still lack in diversity, and thus terminal protection relying on steric hindrance is restricted in designing nucleic acid-based analytical systems. In this work, resistance to exonuclease is testified by group modification or backbone replacement, and the 3′-phosphate group (P) reveals the strongest exonuclease I-resistant capability. Due to the substrate specificity of enzymatic catalysis, this 3′-P protection works in a “direct mode”. By introducing DNA templated copper nanoparticles, an alkaline phosphatase assay is performed to confirm the 3′-P protection. To display the application of this novel terminal protection, a multifunctional DNA is designed to quantify the model circulating microRNA (hsa-miR-21-5p) in serums from different cancer patients. According to our data, hsa-miR-21-5p-correlated cancers can be evidently distinguished from non-correlated cancers. Meanwhile, the effect of chemotherapy and radiotherapy on breast cancer is evaluated from the perspective of hsa-miR-21-5p residue in serums. Since greatly reducing the limitations of DNA design, this P-induced terminal protection can be facilely integrated with other DNA manipulations, thereby constructing more advanced biosensors with improved analytical performances for clinical applications.

## Abstract 2

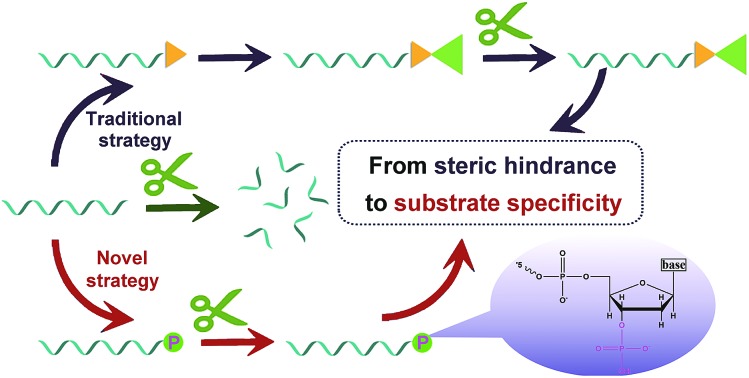
Phosphate group-induced DNA terminal protection is studied, and employed to sensitively detect circulating microRNA in patient serums.

## Introduction

MicroRNAs (miRNAs) are small, endogenous, noncoding RNAs found in diverse organisms, and play key roles in cell development, proliferation, differentiation and apoptosis.^1^–^4^ Recent evidence has shown that miRNAs can repress the expression of important cancer-related genes, thus being considered as new biomarkers.^5^–^7^ In particular, some miRNAs designated as circulating miRNAs (cmiRNAs) exist in various biological fluids like serum and plasma,^8^ exhibiting superiority in providing abundant information for diagnosis and treatment of cancer. By testing cmiRNAs in fluids from patients, cancer can be monitored in a noninvasive manner. However, the tough challenges of cmiRNA quantification are low abundance and high interference. That is to say, the amount of cmiRNAs in fluids is very low, whereas the interference is quite high. Meanwhile, the difference of cmiRNA expression between healthy persons and patients is small. Accordingly, the methodologies of cmiRNA detection have to confront the high demands of sensitivity, accuracy, and anti-interference. Currently, main strategies for cmiRNA detection are quantitative reverse transcription polymerase chain reaction,^9^ microarray-based hybridization methods,^10^ and high-throughput sequencing.^11^ These methods are implemented by base pairing-based molecular recognition, which ensures the accuracy of the detection methods, but poor sensitivity and low anti-interference impede these methods from achieving direct cmiRNA detection in biological fluids. To address these limitations, exploring novel bioanalytical principles based on nucleic acids is a promising way, thereby realizing direct cmiRNA detection in raw fluids.

Apart from molecular recognition, DNA is endowed with some other functions relying on its intrinsic features. DNA can work as templates for the preparation of different metallic nanomaterials, enabling DNA-templated metallic nanoparticles to be the majority of biomaterial-based nanostructures. Owing to the environmental friendliness of copper ions, facile preparation, and outstanding optical properties, newly emerging DNA-templated copper nanoparticles (CuNPs) reveal great potential in constructing a variety of fluorescence biosensors.^12^,^13^ However, signal amplification is normally absent in CuNP-based analytical systems. At present, enzymatic catalysis is an attractive approach to actualize signal amplification, because almost all of the behaviors of DNA, including degradation,^14^–^16^ extension,^17^,^18^ connection and specific recognition,^19^–^24^ are manipulated under the guidance of corresponding enzymes. Hence, a great deal of enzymatic catalysis has been employed in DNA-based biosensors. In particular, duplex-specific nuclease (DSN)-assisted target recycling is an ideal amplification strategy for miRNA detection.^25^ Considering its close relationship with nanomaterials and enzymes, DNA may play a central role in combining multiple functions, such as integrating enzymatic catalysis-induced signal amplification with DNA-templated nanomaterials.

Manipulation of enzymatic catalysis is of great importance in constructing biosensors. One successful paradigm is terminal protection of DNA, on which some pioneering analytical methods have been designed. For example, single stranded DNA (ssDNA) modified with folate or β-indole acetic acid can avoid the digestion of exonuclease I (exo I) and T7 exonuclease, when small molecule moieties are bound to the folate receptor or its antibody.^26^,^27^ Similarly, effect of exonuclease on biotin-linked DNA is impeded by forming biotin–streptavidin affinity pairing.^28^,^29^ One special example is that ATP can protect DNA by interacting with its aptamer.^30^ Although terminal protection has been developed to be a powerful tool, it can be found that the aforementioned methods all work depending on the steric hindrance caused by the interactions between small molecules and corresponding proteins or aptamers. This is an “indirect mode”, meaning activities of enzymes are regulated by preventing the contact of enzyme and its substrate (DNA). Even though the above approaches have been applied in designing biosensors as a proof of concept, the “indirect mode” inevitably suffers from a number of limitations, *e.g.*, complicated modification and sophisticated operation. More importantly, reported small molecule–protein interactions, such as biotin–streptavidin, are rare to date, which greatly limits the applications of this indirect terminal protection in detection of other biomolecules. In consideration of the specificity of enzymatic catalysis, we believe that a slight modification of DNA may also effectively achieve terminal protection, which is a “direct mode” superior to the “indirect mode”. Distinct from indirect terminal protection, direct terminal protection introduces minimal interference to the whole DNA. Accordingly, DNA may be protected against exonuclease while maintaining other functions.

To pursue direct terminal protection of DNA, in this work, we selected phosphate group (P), biotin, DNA, RNA, and LNA modified poly(30T) sequences (*i.e.*, 30T-P, 30T-biotin, 30T-DNA, 30T-RNA, and 30T-LNA). If being kept intact, these poly(30T) sequences can form CuNPs that emit intense fluorescence. However, in the presence of exo I, some of these poly(30T) sequences may be digested, leading to the difference of fluorescence intensity. By comparison of the diversities of fluorescence intensity, it is found that 30T-P exhibits the strongest resistance to exo I, suggesting that 3′-P can effectively protect DNA from digestion of exo I. Meanwhile, the 3′-P protection was further identified with alkaline phosphatase (ALP). Experimental results demonstrate that terminal protection of 30T-P will be abolished in accompany with dephosphorylation caused by ALP, which was employed in fabricating a fluorescence ALP biosensor. Besides, we also designed a novel DNA with the additional function of enzymatic catalysis-caused signal amplification. Since there is a complementary sequence of hsa-miR-21-5p in the middle of the DNA, 3′-P will be separated from the poly(30T) sequence in the presence of DSN and target hsa-miR-21-5p. Without the protection of 3′-P, the poly(30T) sequence will be digested by exo I, resulting in the decline of fluorescence. Benefitting from the 3′-P protection and DSN-caused signal amplification, the hsa-miR-21-5p biosensor reveals excellent sensitivity and anti-interference. More importantly, we collected 80 serums from patients with different cancers as well as treated patients. Hsa-miR-21-5p in different serums is determined with our biosensor, and the difference of hsa-miR-21-5p from different cancer patients is evidently indicated. Besides, both chemotherapy and radiotherapy can induce a decrease of hsa-miR-21-5p in serums of breast cancer patients, and chemotherapy reveals better treatment effect by judging the diminution of hsa-miR-21-5p, suggesting the value of this biosensor in diagnosing and monitoring cancers.

## Results and discussion

### Investigation of direct terminal protection against exo I

**Fig. 1:**
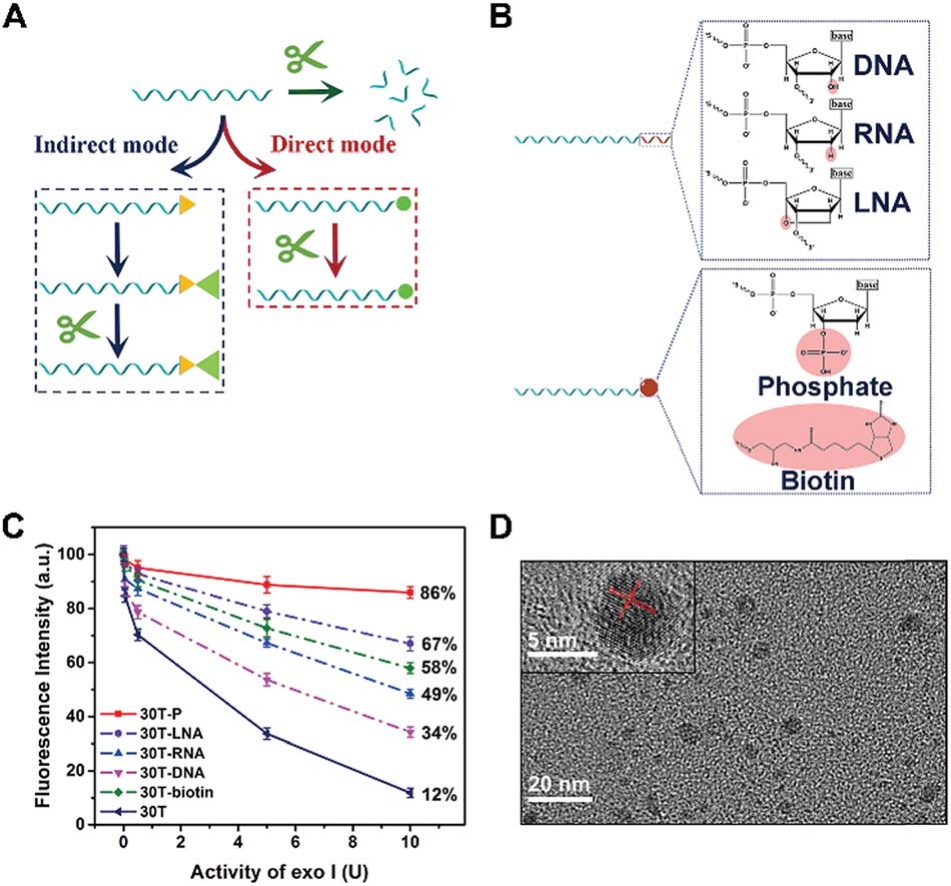
(A) Two strategies for preventing DNA from digestion of exo I by terminal protection. The strategy indicated by the blue dotted frame is the “indirect mode”, while that indicated by the red dotted frame is the “direct mode”. Green arrow represents that DNA is digested by exo I because of the lack of terminal protection. (B) The structural formulae of different modifications at the 3′ terminal of DNA. (C) Effect of exo I on the fluorescence intensity of CuNPs prepared with different DNA templates. (D) HRTEM image of 30T-P-templated CuNPs. Inset: Enlargement of a single CuNP.

Considering that enzymatic catalysis is substrate-specific, we anticipated to inhibit enzymatic catalysis by modifying the substrate (DNA) rather than inducing steric hindrance, which is a “direct-mode” and different from traditional terminal protection (Fig. 1A). By analyzing the structures of nucleic acids, we believe group modification and backbone replacement are possible ways to achieve direct terminal protection. In this work, P, biotin, DNA, RNA, and LNA are linked at the 3′ terminal of the poly(30T) sequence (Fig. 1B). The poly(30T) sequence is a suitable template to synthesize CuNPs, and as expected, all modified poly(30T) sequences in this work can prepare CuNPs with intense fluorescence emission. However, in the presence of exo I, the diversity of fluorescence intensity occurs, reflecting different resistance of these poly(30T) sequences to exo I (Fig. 1C). In general, compared with 30T, 30T-DNA, 30T-RNA, 30T-LNA, 30T-biotin, and 30T-P all display terminal protection to different degrees, demonstrating our assumption that terminal protection can be achieved in a “direct mode”. Specifically, different modifications result in varying resistance in the order of P > LNA > biotin > RNA > DNA. For 30T-P, the majority (86%) of fluorescence can be retained even at a high concentration of exo I. Since P is a small group compared with nucleic acids and phosphorylation of DNA is common, we speculated that besides terminal protection, P may induce limited other influences on the whole DNA sequence, which is desirable in indirect terminal protection. From the perspective of morphology, 30T-P-templated CuNPs are monodisperse with a mean diameter of 3.35 nm (Fig. 1D and S1†), which is consistent with 30T-templated CuNPs. In addition, the crystallinity of CuNPs is not affected by 3′-P, since the crystal lattice structure can be clearly observed. As shown in the inset of Fig. 1D, the lattice spacing of the CuNPs is 2.05 Å, which corresponds to the (111) plane of the fcc phase of copper.^31^ From the perspective of fluorescence behaviors, 30T-P-templated CuNPs and 30T-templated CuNPs possess similar emission spectra (maximum emission at 650 nm), and the effect of 3′-P on fluorescence intensity is negligible (Fig. S2†). All these results verify that 3′-P has no impact on the function of the poly(30T) sequence. In combination with the simplicity of phosphorylation, effectiveness of exo I-resistant capability, and limited influences on other DNA sequences, modification of 3′-P is an ideal way to realize direct terminal protection.

### Identification of P-induced terminal protection using an ALP assay

**Fig. 2:**
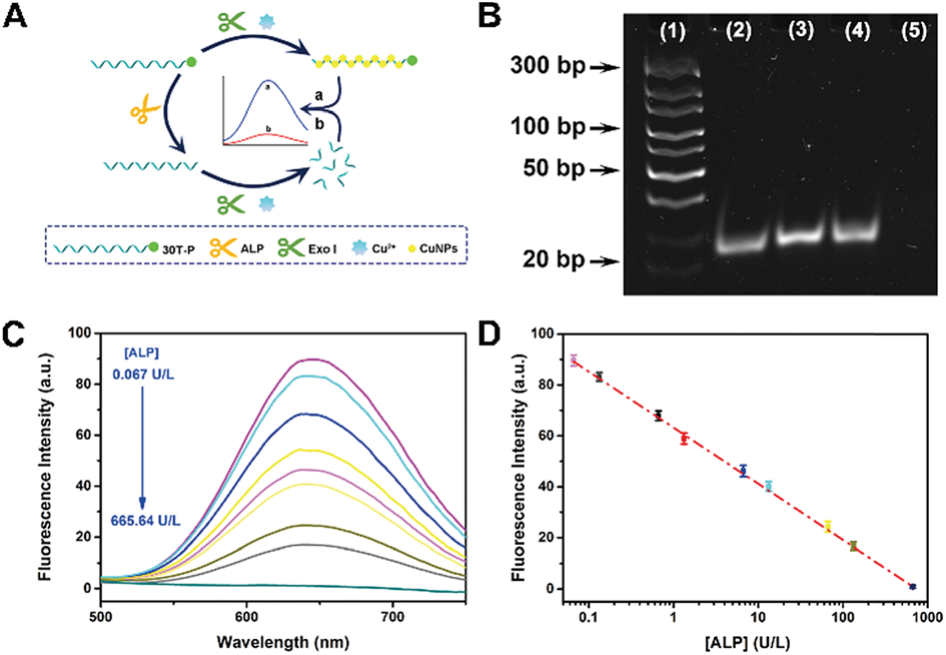
(A) Schematic illustration of an ALP assay based on 30T-P-templated CuNPs and P-induced terminal protection. (B) PAGE image of different samples stained with CuNPs. Lane (1): DNA ladder; lane (2): 30T-P; lane (3): 30T-P and ALP; lane (4): 30T-P and exo I; lane (5): 30T-P, ALP, and exo I. (C) Fluorescence spectra of 30T-P-templated CuNPs. 30T-P is pretreated with different concentrations of ALP. (D) The calibration curve of fluorescence intensity *versus* the concentration of ALP (0.067 to 665.64 U L^–1^).

To further evidence the P-induced terminal protection, an ALP assay was designed on account of 30T-P-templated CuNPs. ALP is an enzyme that can hydrolyze P from the substrate. ALP is an important indicator of health, and the concentration of ALP in serum is highly associated with some hepatic diseases. ALP can hydrolyze P from the substrate. By treatment with ALP, 30T-P can be changed to 30T. Therefore, ALP-treated 30T-P will lose the capability of preventing digestion of exo I. Since only intact poly(30T) sequences can form fluorescent CuNPs, 3′-P protection can be confirmed by comparing the changes of fluorescence (Fig. 2A). The feasibility of the ALP assay is validated by polyacrylamide gel electrophoresis (PAGE) and circular dichroism (CD) measurement. To reveal the 3′-P protection clearly, the amount of exo I used in the ALP assay was first optimized. When the amount of exo I is higher than 10 U, the intensity between 30T-P-templated CuNPs and 30T-templated CuNPs tends to be maximum (Fig. S3†). Therefore, 10 U is chosen as the optimal amount of exo I for the following studies. As shown in Fig. 2B, an apparent band representing 30T-P is obtained in lane (2) of the PAGE image. If 30T-P is treated with either ALP or exo I, a similar band can be evidently observed (lane (3) or lane (4)), reflecting that a single enzyme has no substantive effect on 30T-P. However, if 30T-P is treated with both ALP and exo I, no band can be found in lane (5), indicating that 30T-P is digested by exo I in the case of removing 3′-P by ALP. The results from the PAGE image are in good agreement with that from CD spectra (Fig. S4†). All these data evidence that 3′-P protection can resist the digestion of exo I, and can be eliminated by ALP.

Since elimination of 3′-P protection by ALP can be characterized by the fluorescence of CuNPs, this ALP assay is feasible for the ALP detection. With the increase of concentration of ALP ([ALP]), more poly(30T) sequences are obtained from the dephosphorylation of 30T-P, leading to fluorescence attenuation caused by exo I (Fig. 2C). As can be seen in Fig. 2D, fluorescence intensity at 650 nm (*F*_650 nm_) as a function of [ALP] displays an excellent linearity in the range from 0.067 to 665.64 U L^–1^. The regression equation is *F*_650 nm_ = 63.21 – 22 lg[ALP] (U L^–1^) with a correlation coefficient of 0.9967. The detection limit of this method is 0.03 U L^–1^ (S/N = 3). In comparison with other ALP biosensors, this bioanalytical approach reveals advantages of high sensitivity and a wide detection range (Table S1†), which should be attributed to the reliability of 3′-P protection.

### Fabrication of the hsa-miR-21-5p biosensor *via* P-induced terminal protection and DSN-assisted target recycling

**Fig. 3:**
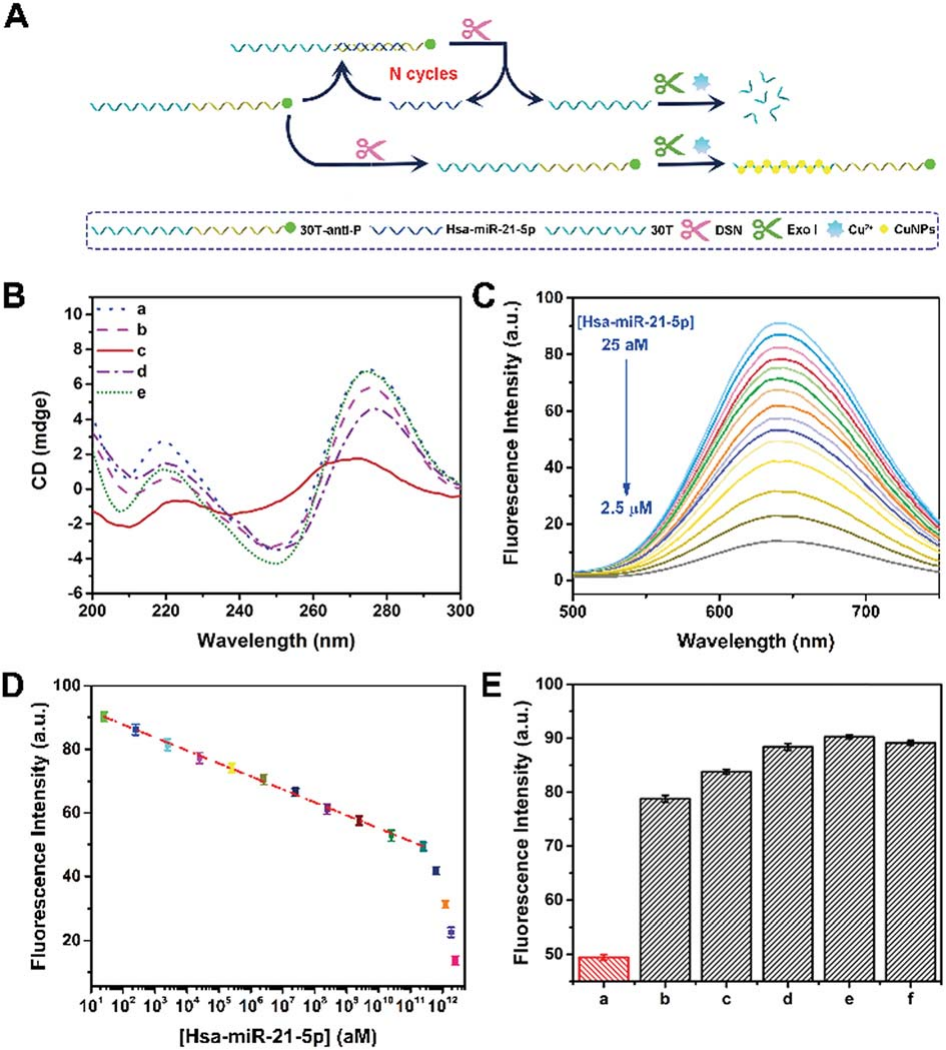
(A) Schematic illustration of a fluorescence hsa-miR-21-5p biosensor based on P-induced terminal protection and DSN-assisted target recycling. (B) CD spectra of (a) 30T-antiRNA-P and hsa-miR-21-5p, (b) 30T-antiRNA-P, hsa-miR-21-5p, and DSN, (c) 30T-antiRNA-P, hsa-miR-21-5p, DSN, and exo I, (d) 30T-antiRNA-P, DSN, and exo I, and (e) 30T-antiRNA-P, hsa-miR-21-5p, and exo I. (C) Fluorescence spectra of 30T-antiRNA-P-templated CuNPs. 30T-antiRNA-P is treated with the different [hsa-miR-21-5p] in the presence of DSN and exo I. (D) The dependence of fluorescence intensity on [hsa-miR-21-5p] in the range from 25 aM to 2.5 μM. (E) *F*_650nm_ of 30T-anitiRNA-P-templated CuNPs. 30T-anitiRNA-P is treated with (a) hsa-miR-21-5p, (b) 1-mis-hsa-miR-21-5p, (c) 3-mis-hsa-miR-21-5p, (d) f-mis-hsa-miR-21-5p, (e) hsa-miR-141-3p, and (f) hsa-let-7d-5p in the presence of DSN and exo I. The concentration of different miRNA is the same (250 nM).

To confirm the applicability of this P-induced terminal protection, a multi-functional DNA was designed to detect hsa-miR-21-5p with the help of DSN-assisted target recycling. The DNA consists of three parts, *i.e.*, 3′-P for terminal protection, the anti-miRNA sequence for miRNA recognition, and the poly(30T) sequence for CuNP preparation. As illustrated in Fig. 3A, in the absence of target cmiRNA (hsa-miR-21-5p), P at the 3′ terminal protects the DNA against exo I. Consequently, the poly(30T) sequence can form CuNPs, and strong fluorescence is obtained. However, in the presence of hsa-miR-21-5p, RNA forms a DNA–RNA heteroduplex by hybridization with the anti-miRNA sequence. DSN is able to selectively cleave DNA in DNA–RNA heteroduplexes, while keeping the RNA intact.^32^ As a result, RNA is released and used to form another heteroduplex that can be cleaved in the next round of DSN-assisted target recycling.^33^ In accompany with the cleavage, P at the 3′ terminal of DNA is separated from the poly(30T) sequence, causing the deprivation of terminal protection. Accordingly, without the 3′-P protection, the poly(30T) sequence is digested by exo I, causing the changes of fluorescence intensity that can be used to quantify hsa-miR-21-5p. To be noted, DSN-assisted target recycling in this system results in signal amplification, which is of high value in measuring cmiRNAs, biological analytes that are in low abundance in biological fluids. In comparison with exonuclease III (exo III)-assisted target recycling, the amplification strategy used in this work avoids the usage of auxiliary strands and interference of RNase H activity of exo III, thus improving the performances of the biosensor.^34^ CD spectra may provide detailed information on the conformational properties of DNA and RNA.^35^ As shown in Fig. 3B, there are two positive peaks at 219 nm and 275 nm and a negative peak at 250 nm in the CD spectrum of DNA–RNA heteroduplexes (curve a).^36^ Addition of DSN causes the cleavage of DNA in DNA–RNA heteroduplexes, resulting in the decrease of positive peaks (curve b). Since DNA is protected by terminal P, addition of exo I does not lead to obvious changes of DNA–RNA heteroduplexes (curve e). If DSN and exo I were both added, a totally different curve was obtained (curve c), demonstrating the destruction of DNA–RNA heteroduplexes. However, if target cmiRNA is absent, the conformation of DNA would not be destroyed by DSN and exo I (curve d). The remarkable difference indicates that the presence of target cmiRNA is crucial to remove the 3′-P protection. Another piece of direct evidence is provided by PAGE analysis (Fig. S5†), which agrees well with CD measurements. In conclusion, results from PAGE and CD characterization are consistent, confirming that the multi-functional DNA can sense target cmiRNA (hsa-miR-21-5p) with the help of P-induced terminal protection and DSN-assisted target recycling.

This fluorescence method was further utilized to measure the concentration of hsa-miR-21-5p ([hsa-miR-21-5p]). With the increase of hsa-miR-21-5p, more DNA in DNA–RNA heteroduplexes is cleaved by DSN, releasing target hsa-miR-21-5p and 3′-P. Without the protection of 3′-P, the poly(30T) sequence will be digested by exo I, and the fluorescence of CuNPs gradually becomes weak because of the lack of templates. To be noted, released hsa-miR-21-5p may trigger DSN-assisted target recycling again, and thus the signal of this sensing system is significantly amplified. As shown in Fig. 3C, a dramatic fluorescence decrease is observed with the increase of [hsa-miR-21-5p]. In the range from 25 aM to 250 nM, logarithm of [hsa-miR-21-5p] exhibits a good linear relationship with *F*_650 nm_ (Fig. 3D). The calibration equation is *F*_650 nm_ = 95.95 – 4.08 lg[hsa-miR-21-5p] (*R* = 0.998), and the calculated detection limit is 16 aM (S/N = 3). Thanks to the strong fluorescence of CuNPs and signal amplification of target recycling, the linear range and detection limit are better than or at least comparable to those of previously reported biosensors for hsa-miR-21-5p detection (Table S2†). It is known that [hsa-miR-21-5p] in human serum is at the femtomolar level.^37^ Although the method works in a “signal off” manner, the analytical performances of this biosensor can ensure its applications in practical samples. To be noted, this biosensor is not restricted to cmiRNA. Target RNAs with longer or shorter sequences can also be detected by our method (Fig. S6†).

The selectivity of the proposed method was further confirmed by other five miRNAs, including 1-mis-hsa-miR-21-5p, 3-mis-hsa-miR-21-5p, f-mis-hsa-miR-21-5p, hsa-miR-141-3p, and hsa-let-7d-5p. By replacing hsa-miR-21-5p with these miRNAs, the fluorescence of DNA-templated CuNPs is distinctly enlarged (Fig. 3E), manifesting the satisfactory selectivity of this cmiRNA biosensor. The specificity of this biosensor was further testified using 5′-hsa-miR-21-5p and 3′-hsa-miR-21-5p (5′ or 3′ addition isomiRs) (Fig. S7†). These results evidence that target hsa-miR-21-5p initiates the cleavage of DSN by forming DNA–RNA heteroduplexes that are then digested by exo I. In contrast, other miRNAs, even one-base mismatch miRNA (1-mis-hsa-miR-21-5p), cannot cause the separation of 3′-P and the poly(30T) sequence, thus maintaining strong fluorescence due to 3′-P protection. The extraordinary selectivity of this method should be attributed to the high specificity of DSN-assisted target recycling and efficiency of P-induced terminal protection. Considering the complexity of serums, outstanding selectivity is of great value in cmiRNA detection.

### Detection of hsa-miR-21-5p in serums from cancer patients

**Fig. 4:**
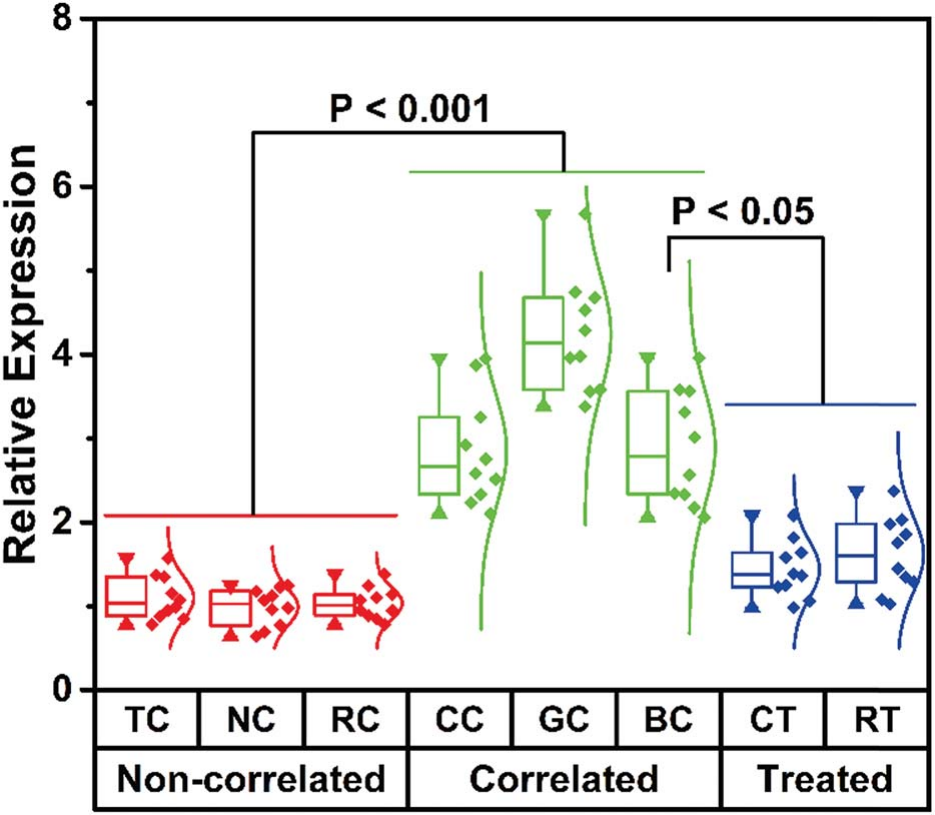
The histogram for hsa-miR-21-5p expression in serums from different cancer patients.

The main aim of this analytical system is to achieve direct cmiRNA detection in biological fluids. Having identified the advantageous sensitivity and superior selectivity of this biosensor, we attempted to challenge this biosensor using serums from cancer patients. We collected 60 samples from different cancer patients including thyroid cancer (TC), nasopharynx cancer (NC), renal cell cancer (RC), colorectal cancer (CC), gastric cancer (GC), and breast cancer (BC), as well as 20 samples from BC patients who underwent chemotherapy (CT) and radiotherapy (RT). According to the data from our proposed biosensor, low hsa-miR-21-5p expression is found in serums of TC, NC, and RC, whereas high hsa-miR-21-5p expression is observed in CC, GC, and BC (Fig. 4). That is to say, some of the cancers (like the latter three kinds of cancers) are hsa-miR-21-5p-correlated cancers, and there is a statistically significant difference between correlated cancers and non-correlated cancers (*P* < 0.001), which is in accordance with other studies.^38^,^39^ By setting the mean levels of non-correlated cancers as 1, the mean levels of correlated cancers are much higher (2.8, 4.2, and 2.9, respectively). Besides detection of hsa-miR-21-5p in different serums, we also monitored the curative effect of different therapies on correlated cancers. To our knowledge, CT and RT are the most common strategies for BC treatment. From the perspective of hsa-miR-21-5p residue in serums, both CT and RT are effective methods in treating BC, and CT is better for a lower amount of hsa-miR-21-5p in CT-treated patients. It should not be ignored that the mean levels of hsa-miR-21-5p in treated patients (1.4 and 1.6) are still higher than those of non-correlated patients. By directly evaluating hsa-miR-21-5p expression in serums, patients with correlated cancers and non-correlated cancers can be evidently distinguished using our method, and the effect of different therapies can be accurately monitored, indicating the great potential of this method in diagnosis and postoperative observation of cancers.

## Conclusions

In this work, five types of modified poly(30T) sequences have been selected to compare their exo I-resistant capability. Relying on the fluorescence of CuNPs using poly(30T) sequences as templates, it is found that 30T-P exhibits the strongest resistance to exo I, and thus a kind of direct terminal protection is developed, which is different from the previously reported indirect terminal protection depending on steric hindrance. Since ALP can dephosphorylate the terminal P of DNA, the P-induced terminal protection has been identified by an ALP assay, and has been further used to detect ALP. To broaden the applications of this P-induced terminal protection, a multi-functional DNA that can execute terminal protection, RNA recognition, and template function, is designed to fabricate a fluorescence cmiRNA biosensor. Besides, to achieve signal amplification, DSN-assisted target recycling is also integrated into the bioanalytical approach. By choosing hsa-miR-21-5p as a model cmiRNA, the proposed biosensor exhibits high sensitivity and selectivity for hsa-miR-21-5p detection. More importantly, hsa-miR-21-5p in serums from cancer patients can be directly quantified by this method, which maximizes the significance of cmiRNA detection. According to the data from our biosensor, hsa-miR-21-5p-correlated cancers can be facilely distinguished from non-correlated ones, and CT is a better treatment than RT for BC patient. Overall, benefitting from the properties of the “direct mode”, this P-induced terminal may reveal great potential in designing advanced analytical systems for clinical applications.

## Experimental

### Chemicals and materials

Alkaline phosphatase (ALP) was purchased from Shanghai Sangon Biological Engineering Technology & Services Co., Ltd. (Shanghai, China). Copper chloride (CuCl_2_), ascorbic acid (Vc), 3-(*N*-morpholino)propanesulfonic acid (MOPS), tris(hydroxymethyl)aminomethane hydrochloride (Tris–HCl), boric acid (H_3_BO_3_), and ethylene diamine tetraacetic acid (EDTA) were obtained from Sigma-Aldrich Co., Ltd. (Shanghai, China). Duplex-specific nuclease (DSN) was purchased from Newborn Co., Ltd. (Shenzhen, China). Exonuclease I (exo I), recombinant RNase inhibitor (RRI), and diethyl pyrocarbonate (DEPC)-treated water (RNase-free) were obtained from Takara Biotechnology Co., Ltd. (Dalian, China). Human serums were provided by Jiangsu Cancer Hospital (Nanjing, China), and used with the approval of the ethical committee of Nanjing Normal University. All chemicals used were of analytically pure grade and directly used without further purification.

All oligonucleotides shown in Table 1 were obtained and purified with high performance liquid chromatography by GenScript Biotech. Co., Ltd. (Nanjing, China). DNA and RNA were diluted with DEPC-treated water. CuCl_2_ was dissolved in MOPS (10 mM, pH 7.5) as a stock solution. Other solutions were prepared with ultrapure water (18.2 MΩ cm) obtained from a Milli-Q purification system (Bedford, MA).

**Table 1:** Sequences of oligonucleotides used in this work^*a*^

Name	Sequence from 5′ to 3′
30T-P	*TTTTTTTTTTTTTTTTTTTTTTTTTTTTTTT*(*)
30T-Biotin	*TTTTTTTTTTTTTTTTTTTTTTTTTTTTTTT*(B)
30T-LNA	*TTTTTTTTTTTTTTTTTTTTTTTTTTTTTTT*( 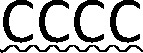 )
30T-RNA	*TTTTTTTTTTTTTTTTTTTTTTTTTTTTTTT*( 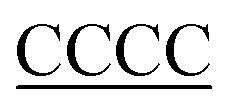 )
30T-DNA	*TTTTTTTTTTTTTTTTTTTTTTTTTTTTTTT*(CCCC)
30T-AntiRNA-P	*TTTTTTTTTTTTTTTTTTTTTTTTTTTTTTT*CAACATCAGTCTGATAAGCTA(*)
hsa-miR-21-5p	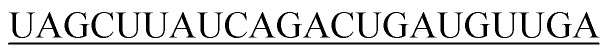
1-mis-hsa-miR-21-5p	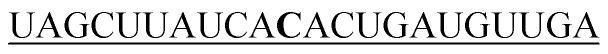
3-mis-hsa-miR-21-5p	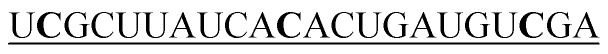
f-mis-hsa-miR-21-5p	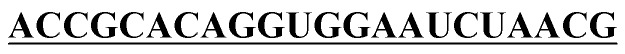
hsa-miR-141-3p	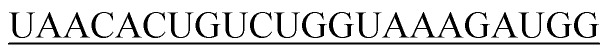
hsa-let-7d-5p	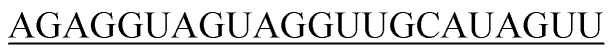
10-R	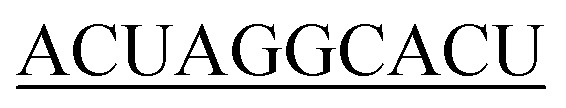
30T-Anti10R-P	*TTTTTTTTTTTTTTTTTTTTTTTTTTTTTT*AGTGCCTAGT(*)
40-R	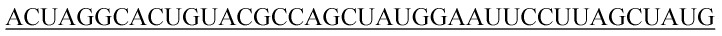
30T-anti40R-P	*TTTTTTTTTTTTTTTTTTTTTTTTTTTTTT*CATAGCTAAGGAATTCCATAGCTGGCGTACAGTGCCTAGT(*)
3′-hsa-miR-21-5p	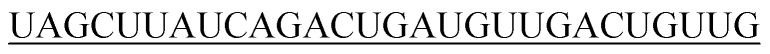
5′-hsa-miR-21-5p	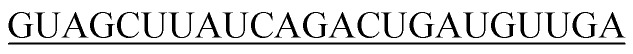

^*a*^The italic sequences are used to prepare CuNPs as templates. The symbol of * represents the modified phosphate group in the 3′ terminal of the sequence, while B represents the modification of biotin. The LNA sequence and RNA sequence are indicated by the wavy line and underline, respectively. Mismatched bases are marked by bold.

### Instruments

Fluorescence measurements were performed on a Fluoromax-4 spectrometer (Horiba, France). CuNPs were excited at 340 nm, and the fluorescence emission spectra were recorded from 500 nm to 750 nm with a 400 nm optical filter. High-resolution transmission electron microscopy (HRTEM, JEOL-2100F, 200 kV) was employed to characterize the morphologies of CuNPs. CD measurements were accomplished using quartz cells with a 1 mm path length (Chirascan, Applied Photophysics, Britain). The experimental parameters were listed as follows: 1 nm bandwidth, 1 nm step size, and the scan range was acquired in the region of 200–300 nm.

### Preparation of DNA-templated CuNPs

DNA-templated CuNPs were synthesized according to our previous studies.^13^ Briefly, DNA was first dissolved in MOPS solution (10 mM, pH 7.5) to form a reaction solution. After that, 10 μL of 1 mM CuCl_2_ and 15 μL of 20 mM Vc were mixed with 275 μL reaction solution, and the mixture was incubated 15 min in the dark. Then, DNA-templated CuNPs were prepared and ready for fluorescence analysis.

### Analysis of P-induced terminal protection

Six types of DNA (30T, 30T-P, 30T-LNA, 30T-RNA, 30T-DNA, and 30T-biotin) were dissolved with a final concentration of 10 μM. Subsequently, different amounts of exo I (from 0 to 10 U) were added into 15 μL DNA solution and incubated at 37 °C for 30 min. Finally, the obtained DNA solution was used to prepare CuNPs.

### ALP assay

First, 2 μL of ALP with different concentrations and 3 μL DEPC-treated water were incubated with 15 μL of 10 μM 30T-antiRNA-P at 37 °C for 90 min. Next, 10 U exo I and 3 μL of 10× buffer were added into the solution and kept at 37 °C for 30 min. Then, the CuNPs were formed by adding CuCl_2_, Vc and MOPS, and the fluorescence intensity of CuNPs was recorded.

### Fabrication of a fluorescence cmiRNA biosensor

Hsa-miR-21-5p was adopted as a model cmiRNA to fabricate a fluorescence biosensor based on P-induced terminal protection and DSN-assisted target recycling. To achieve DSN-assisted target recycling, 15 μL target cmiRNA with different concentrations, 15 μL of 10 μM 30T-antiRNA-P, 5 μL of 0.1 U μL^–1^ DSN, 2 μL of 5 U μL^–1^ RRI, 6 μL of 10× buffer, and 17 μL DEPC-treated water, were mixed in a volume of 60 μL at 60 °C for 1 h. Then, 10 U exo I was added and incubated at 37 °C for 30 min. Finally, the above solution was used to form CuNPs for fluorescence measurements.

### CD measurements and PAGE analysis

Samples for CD measurements and PAGE analysis were prepared based on the procedure of fluorescence detection, while higher concentrations of nucleic acids and enzymes were needed. The final concentrations of 30T-P, 30T-antiRNA-P, hsa-miR-21-5p, ALP, and DSN were 4.5 μM, 4.3 μM, 4.3 μM, 403 U L^–1^, and 14.3 U mL^–1^, respectively. Staining of the poly(30T) sequence in the PAGE assay was executed according to previous studies.^13^,^40^

### Selectivity assay

To testify its selectivity, the biosensor was challenged with other miRNAs (1-mis-hsa-miR-21-5p, 3-mis-hsa-miR-21-5p, f-mis-hsa-miR-21-5p, hsa-miR-141-3p, and hsa-let-7d-5p) instead of target cmiRNA (hsa-miR-21-5p). Briefly, 15 μL of 1 μM different RNAs, 15 μL of 10 μM 30T-antiRNA-P, 5 μL of 0.5 U μL^–1^ DSN, 2 μL of 5 U μL^–1^ RRI, and 6 μL of 10× buffer were mixed at 60 °C for 1 h. After that, 10 U exo I was incubated with the mixture at 37 °C for 30 min. The following procedure was similar to that of biosensor construction.

### Quantification of hsa-miR-21-5p in serums from patients

Human blood samples obtained from various cancer patients were first coagulated to get the supernate (serums). Subsequently, 15 μL of diluted 1% serums, 15 μL of 10 μM 30T-antiRNA-P, 5 μL of 0.1 U μL^–1^ DSN, 2 μL of 5 U μL^–1^ RRI, and 6 μL of 10× buffer were mixed at 60 °C for 1 h. Then, quantification of hsa-miR-21-5p was accomplished according to the proposed fluorescence biosensor.

## Conflicts of interest

There are no conflicts to declare.

## Supplementary Material

Supplementary information
