# Supplementary material for: Substrate specificity-enabled terminal protection for direct quantification of circulating MicroRNA in patient serums
Source: Chem Sci. 2019 May 1;10(21):5616–23. doi: 10.1039/c8sc05240a (PMC6552989; doi:10.1039/c8sc05240a)
Supplement: Supplementary file 1 [file SC-010-C8SC05240A-s001.pdf]

Electronic Supplementary Material (ESI) for Chemical Science.
This journal is © The Royal Society of Chemistry 2019

Supporting Information

# Substrate Specificity-Enabled Terminal Protection for Direct Quantification of Circulating MicroRNA in Patient Serums

Junyao Li,[a] Wenxin Fu,[a] Zhaoyin Wang,*[a] and Zhihui Dai*[ab]

[a]Jiangsu Collaborative Innovation Center of Biomedical Functional Materials and Jiangsu Key Laboratory of Biofunctional Materials, School of Chemistry and Materials Science, Nanjing Normal University, Nanjing, 210023, P. R. China.

[b]Nanjing Normal University Center for Analysis and Testing, Nanjing, 210023, P. R. China.

*Tel./Fax: +86-25-85891051. E-mail: daizhihuii@njnu.edu.cn (Z. Dai).

## List of contents:

1. Diameter distribution of 30T-P-templated CuNPs (Fig. S1).
2. Effect of exo I on fluorescence of 30T-templated CuNPs and 30T-P-templated CuNPs (Fig. S2).
3. Optimization of exo I in P-induced terminal protection (Fig. S3).
4. Results of CD measurements for the ALP assay (Fig. S4).
5. Comparison of different ALP biosensors (Table S1).
6. Results of PAGE analysis for the hsa-miR-21-5p biosensor (Fig. S5).
7. Comparison of different hsa-miR-21-5p biosensors (Table S2).
8. Effect of RNA length on fluorescence response of CuNPs (Fig. S6).
9. Selectivity of the proposed method in the presence of isoforms (Fig. S7).
10. Related references for supporting information (Reference S1 - S24).

**1. Diameter distribution of 30T-P-templated CuNPs.**

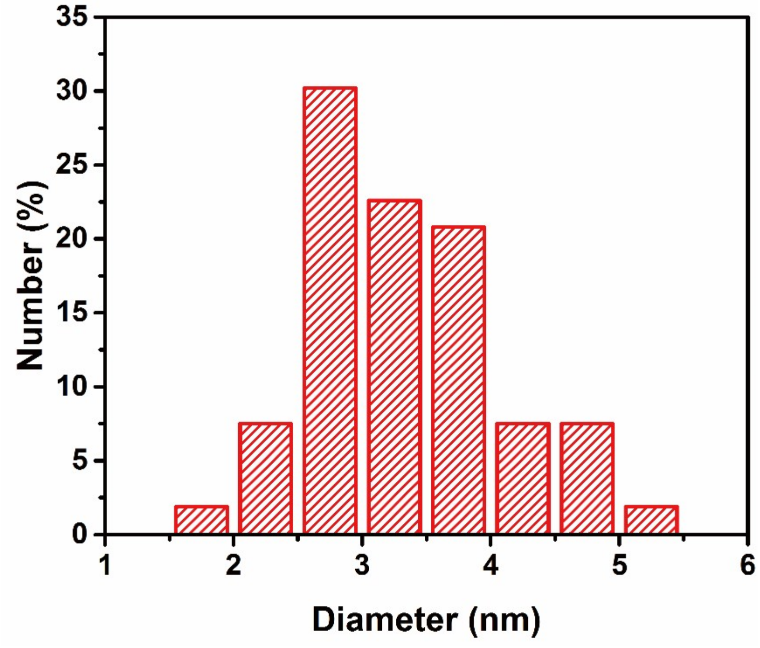


**Fig. S1.** Diameter distribution of 30T-P-templated CuNPs.

**2. Effect of exo I on fluorescence of 30T-templated CuNPs and 30T-P-templated CuNPs.**

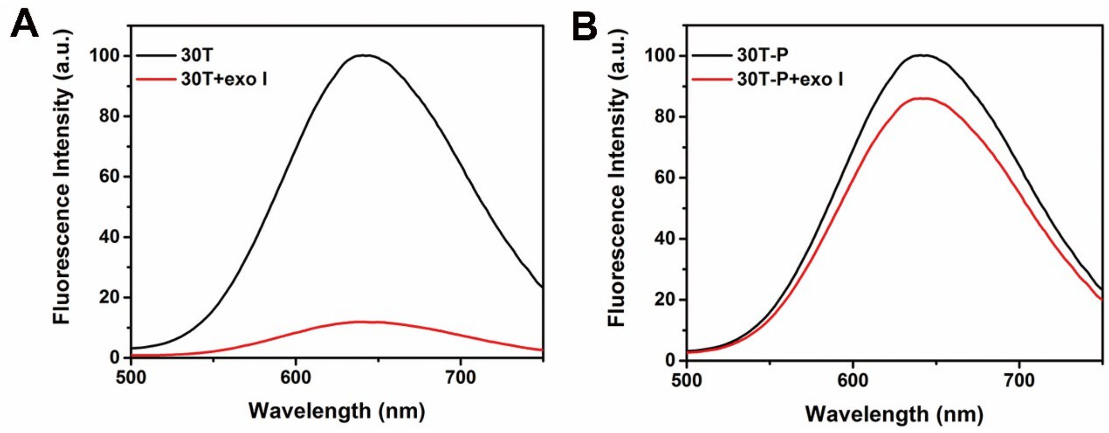


**Fig. S2.** (A) Fluorescence spectra of 30T-templated CuNPs with and without 10 U exo I. (B) Fluorescence spectra of 30T-P-templated CuNPs with and without 10 U exo I.

### 3. Optimization of exo I in P-induced terminal protection.

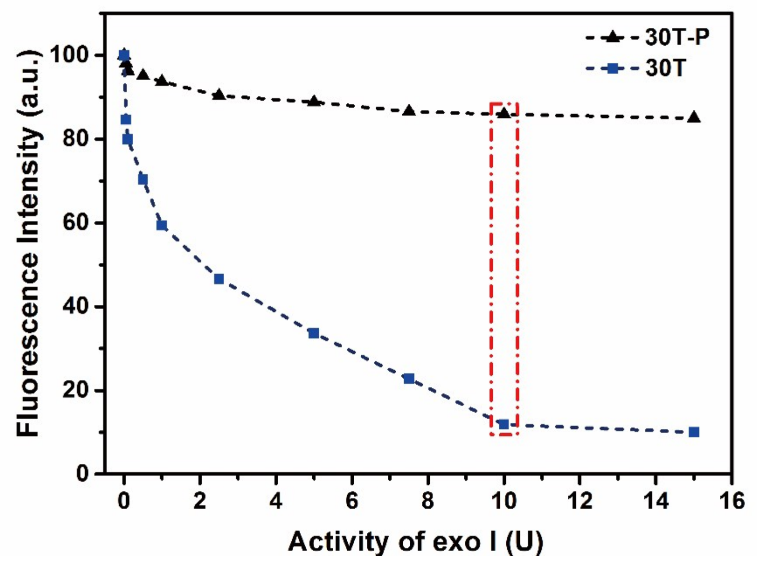


**Fig. S3.** Fluorescence intensity of 30T-templated CuNPs and 30T-P-templated CuNPs as a function of exo I.

## 4. Results of CD measurements for the ALP assay.

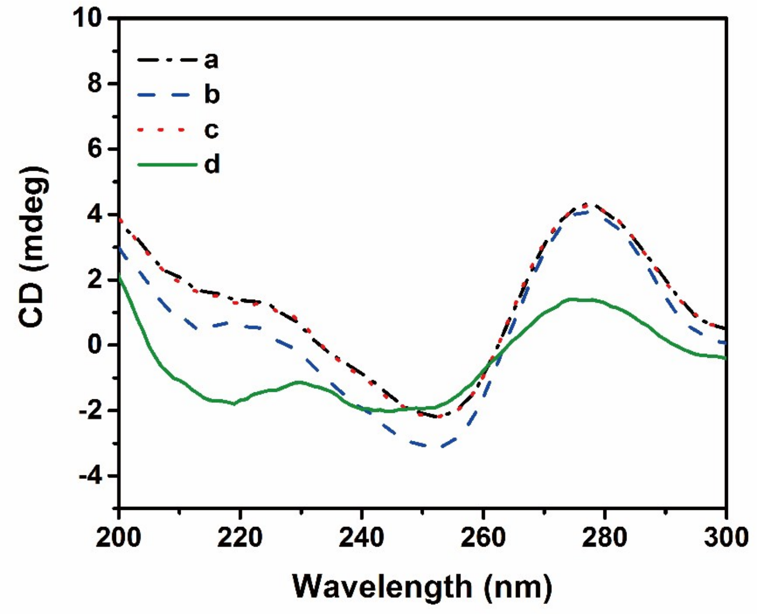


**Fig. S4.** CD spectra of (a) 30T-P, (b) 30T-P and ALP, (c) 30T-P and exo I, and (d) 30T-P, ALP, and exo I.

## 5. Comparison of different ALP biosensors (Table S1).

**Table S1**. Comparison of analytical performances of different ALP biosensors.

| Detection method | Signal probe | Detection limit | Linear range | Ref. |
|---|---|---|---|---|
| **Colorimetry** | Upconversion nanoparticles | 0.065 U/L | 0.08 - 70 U/L | S1 |
| **Colorimetry** | Gold nanoparticle | 0.032 U/L | 0.032 - 100 U/L | S2 |
| **Colorimetry** | Gold/silver core/shell nanorod | 3.3 U/L | 5 - 100 U/L | S3 |
| **Photoelectrochemical** | CdTe quantum dots | 0.15 U/L | 0.2 - 15 U/L | S4 |
| **Chemiluminescence** | MSN@RhB@b-CD@ AMPPD | 0.0748 U/L | 0 - 400 U/L | S5 |
| **Electrochemistry** | Nanoceria particles | 20 U/L | 5000 - 64000 U/L | S6 |
| **Electrochemistry** | Molecular beacon | 0.1 U/L | 0.1 - 10 U/L | S7 |
| **Electrochemiluminescence** | CdSe nanoparticles | 2 U/L | 2 - 25 U/L | S8 |
| **Fluorescence** | Silicon nanoparticle | 0.2 U/L | 0.2 - 30 U/L | S9 |
| **Fluorescence** | Gold nanoclusters | 0.05 U/L | 1 - 200 U/L | S10 |
| **Fluorescence** | Upconversion nanoparticles | 0.032 U/L | 0.08 - 70 U/L | S1 |
| **Fluorescence** | $MoS_2$ quantum dots | 0.1 U/L | 0 - 5 U/L | S11 |
| **Fluorescence** | $MnO_2$ nanosheets | 0.045 U/L | 0.13 - 10 U/L | S12 |
| **Fluorescence** | Copper (II)-based metal-organic frameworks | 0.19 U/L | 1 -34 U/L | S13 |
| **Fluorescence** | Copper nanoparticles | 0.03 U/L | 0.067 - 665.64 U/L | This work |

## 6. Results of PAGE analysis for the hsa-miR-21-5p biosensor.

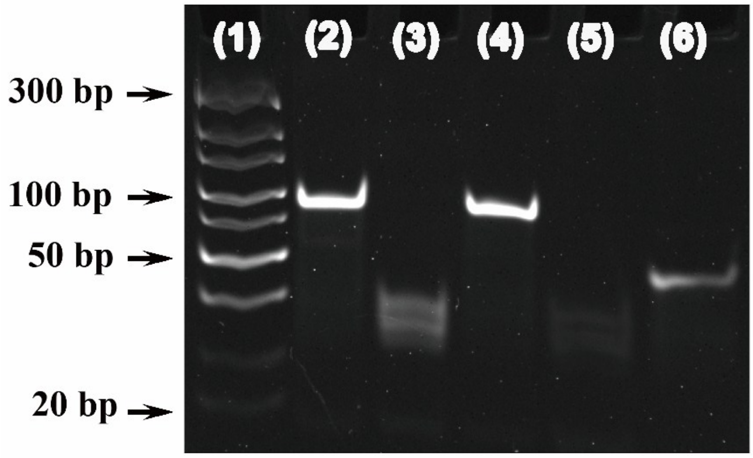


**Fig. S5.** PAGE image of different samples stained by CuNPs. Lane (1): DNA ladder; lane (2): 30T-antiRNA-P and hsa-miR-21-5p; lane (3): 30T-antiRNA-P, hsa-miR-21-5p, and DSN; lane (4): 30T-antiRNA-P, hsa-miR-21-5p, and exo I; lane (5): 30T-antiRNA-P, hsa-miR-21-5p, ALP, and exo I; lane (6): 30T-antiRNA-P, ALP, and exo I.

## 7. Comparison of different hsa-miR-21-5p biosensors.

**Table S2.** Comparison of analytical performances of different hsa-miR-21-5p biosensors.

| Detection method | Signal probe | Detection limit | Linear range | Real sample detection | Ref. |
|---|---|---|---|---|---|
| **Colorimetry** | Graphene/gold-nanoparticle hybrids | 3.2 nM | 10 nM - 980 nM | Human serum | S14 |
| **Electrochemistry** | Silver nanoparticle | 400 aM | 1 fM - 1 nM | Human serum | S15 |
| **Electrochemistry** | Methylene blue modified DNA fuel strand | 1.4 fM | 5 fM - 500 pM | MCF-7 cells and Hela cells | S16 |
| **Electrochemistry** | Gold nanoparticle-decorated $MoS_2$ nanosheet | 450 aM | 10 fM - 1 nM | Human serum | S17 |
| **Electrochemistry** | Gold nanoparticles | 100 pM | 200 pM - 388 nM | Not applicable | S18 |
| **Electrochemiluminescence** | Au nanoparticles functionalized g-$C_3N_4$ NS nanohybrid | 500 aM | 1 fM - 1 nM | HeLa cells | S19 |
| **Electrochemiluminescence** | Gold nanoparticals functionalized reduced graphene oxide | 30 aM | 100 aM - 10 pM | A549 cells | S20 |
| **Impedance** | Biotin-molecular beacon-gold nanoparticles | 300 fM | 1 pM - 1 nM | Human serum | S21 |
| **Fluorescence** | Copper nanoparticles | 100 fM | 1 pM - 1 nM | MCF-7 cells, and A549 cells | S22 |
| **Fluorescence** | Graphene oxide | 47 pM | 47 pM - 16 nM | Hep-2 cells, A549 cells, and HUVEC | S23 |
| **Fluorescence** | AMCA dye | 270 aM | 1 fM - 50 pM | Human serum | S24 |
| **Fluorescence** | Copper nanoparticles | 16 aM | 25 aM - 250 nM | Human serum | This work |

## 8. Effect of RNA length on fluorescence response of CuNPs.

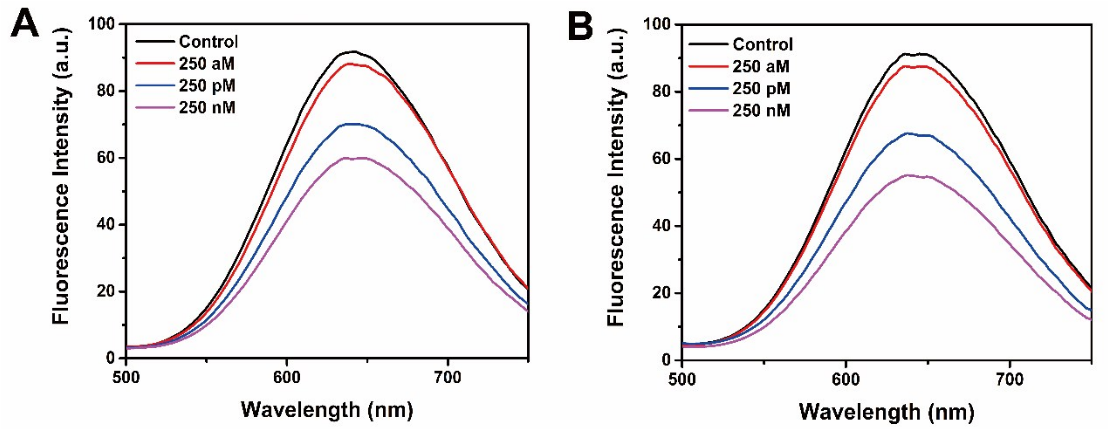


**Fig. S6.** Fluorescence spectra of (A) 30T-anti10R-P-templated CuNPs and (B) 30T-anti10R-P-templated CuNPs. 30T-anti10R-P is treated with the different concentrations of 10-R in the presence of DSN and exo I, while 30T-anti40R-P is treated with the different concentrations of 40-R.

## 9. Selectivity of the proposed method in the presence of isoforms.

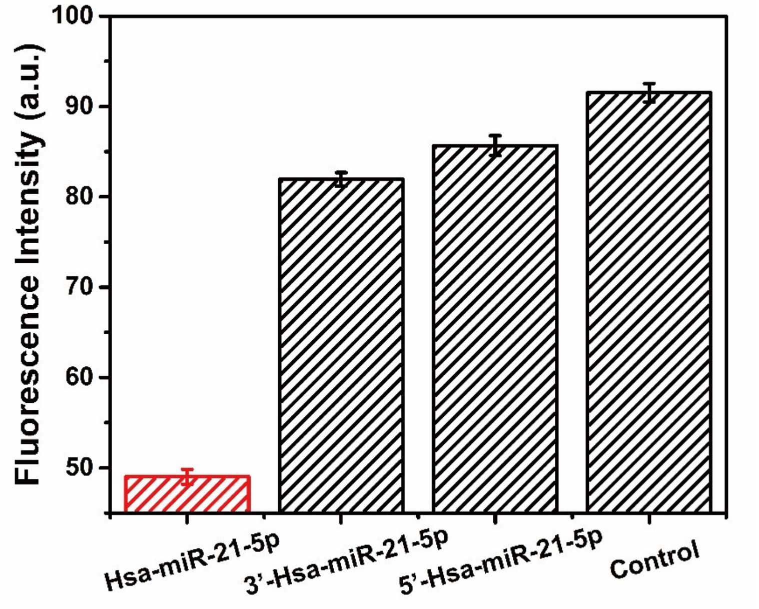


**Fig. S7.** $F_{650nm}$ of 30T-anitiRNA-P-templated CuNPs. 30T-anitiRNA-P is treated with hsa-miR-21-5p, 3'-hsa-miR-21-5p and 5'-hsa-miR-21-5p in the presence of DSN and exo I. The concentration of a single miRNA in this assay is the same (250 nM).

## 10. References


S1. H. Chen, Z. Zhou, Q. Lu, C. Wu, M. Liu, Y. Zhang and S. Yao, *Anal. Chim. Acta*, 2019, **1051**, 160-168.

S2. H. Jiao, J. Chen, W. Li, F. Wang, H. Zhou, Y. Li and C. Yu, *ACS Appl. Mater. Interfaces*, 2014, **6**, 1979-1985.

S3. Z. Gao, K. Deng, X. D. Wang, M. Miro and D. Tang, *ACS Appl. Mater. Interfaces*, 2014, **6**, 18243-18250.

S4. J. Tian, Y. Yang, M. Huang, C. Zhou and J. Lu, *Talanta*, 2019, **196**, 293-299.

S5. X. Liu, N. Fan, L. Wu, C. Wu, Y. Zhou, P. Li and B. Tang, *Chem. Commun.*, 2018, **54**, 12479-12482.

S6. A. Hayat and S. Andreescu, *Anal. Chem.*, 2013, **85**, 10028-10032.

S7. L. Zhang, T. Hou, H. Li and F. Li, *Analyst*, 2015, **140**, 4030-4036.

S8. H. Jiang and X. Wang, *Anal. Chem.*, 2012, **84**, 6986-6993.

S9. J. Sun, T. Hu, C. Chen, D. Zhao, F. Yang and X. Yang, *Anal. Chem.*, 2016, **88**, 9789-9795.

S10. M. I. Halawa, W. Gao, M. Saqib, S. A. Kitte, F. Wu and G. Xu, *Biosens. Bioelectron.*, 2017, **95**, 8-14.

S11. Y. Zhong, F. Xue, P. Wei, R. Li, C. Cao and T. Yi, *Nanoscale*, 2018, **10**, 21298-21306.

S12. W. Na, N. Li and S. Xingguang, *Sens. Actuators, B*, 2018, **274**, 172-179.

S13. C. Wang, J. Gao, Y. Cao and H. Tan, *Anal. Chim. Acta*, 2018, **1004**, 74-81.

S14. H. Zhao, Y. Qu, F. Yuan and X. Quan, *Anal. Methods*, 2016, **8**, 2005-2012.

S15. P. Miao, B. D. Wang, X. F. Chen, X. X. Li and Y. G. Tang, *ACS Appl. Mater. Interfaces*, 2015, **7**, 6238-6243.

S16. K. Shi, B. T. Dou, C. Y. Yang, Y. Q. Chai, R. Yuan and Y. Xiang, *Anal. Chem.*, 2015, **87**, 8578-8583.

S17. S. Su, W. Cao, W. Liu, Z. Lu, D. Zhu, J. Chao, L. Weng, L. Wang, C. Fan and L. Wang, *Biosens. Bioelectron.*, 2017, **94**, 552-559.

S18. J. Mandli, H. Mohammadi and A. Amine, *Bioelectrochemistry*, 2017, **116**, 17-23.

S19. Q. M. Feng, Y. Z. Shen, M. X. Li, Z. L. Zhang, W. Zhao, J. J. Xu and H. Y. Chen, *Anal. Chem.*, 2016, **88**, 937-944.

S20. Y. Q. Yu, J. P. Wang, M. Zhao, L. R. Hong, Y. Q. Chai, R. Yuan and Y. Zhuo, *Biosens. Bioelectron.*, 2016, **77**, 442-450.

S21. S. Azzouzi, W. C. Mak, K. Kor, A. P. F. Turner, M. Ben Ali and V. Beni, *Biosens. Bioelectron.*, 2017, **92**, 154-161.

S22. B. Z. Chi, R. P. Liang, W. B. Qiu, Y. H. Yuan and J. D. Qiu, *Biosens. Bioelectron.*, 2017, **87**, 216-221.

S23. S. J. Zhen, X. Xiao, C. H. Li and C. Z. Huang, *Anal. Chem.*, 2017, **89**, 8766-8771.

S24. H. S. Yin, B. C. Li, Y. L. Zhou, H. Y. Wang, M. H. Wang and S. Y. Ai, *Biosens. Bioelectron.*, 2017, **96**, 106-112.
